# Editorial: Recent advances in translational neurovascular and cerebroprotection research

**DOI:** 10.3389/fnins.2026.1861792

**Published:** 2026-05-15

**Authors:** Johannes Boltze, Peiying Li, Ann M. Stowe, Marietta Zille, Nikolaus Plesnila

**Affiliations:** 1 School of Life Sciences, University of Warwick, Coventry, United Kingdom; 2 Department of Anesthesiology, Renji Hospital, Shanghai Jiao Tong University School of Medicine, Shanghai, China; 3 Key Laboratory of Anesthesiology, Shanghai Jiao Tong University, Ministry of Education, Shanghai, China; 4 Clinical Research Center, Renji Hospital, Shanghai Jiao Tong University School of Medicine, Shanghai, China; 5 Department of Neuroscience, University of Kentucky, Lexington, KY, United States; 6 Department of Neurology, University of Kentucky, Lexington, KY, United States; 7 Department of Pharmaceutical Sciences, Division of Pharmacology and Toxicology, University of Vienna, Vienna, Austria; 8 Institute for Stroke and Dementia Research, Klinikum der Universität München and Ludwig Maximilian University (LMU) Munich, Munich, Germany; 9 Munich Cluster for Systems Neurology (SyNergy), Munich, Germany

**Keywords:** brain stimulation, cerebral hemorrhage, cerebral ischemia, cerebrovascular disease, dementia, neurodegeneration, neuroimaging, neuroprotection

Recent research efforts have resulted in an increasing understanding of the pathological mechanisms underlying neurovascular and neurodegenerative disorders, yet disease-modifying, causal therapeutic interventions are still lacking. However, there have been advances in defining new cerebroprotection approaches for numerous diseases, and novel diagnostic and therapeutic concepts are currently assessed in translational studies. This Research Topic aims to summarize such exciting novel concepts as well as recent advances. Articles were collected in collaboration with the 11th International Symposium on Neuroprotection & Neurorepair (ISN&N) which took place in November 2024 in Potsdam, Germany. Moreover, the Research Topic was open to unsolicited submissions from the global scientific community, provided topical fit. Eleven articles written by 91 authors are featured in this research topic, comprising preclinical and clinical original contributions as well as review articles.

## Advances in preclinical and translational research

Ferroptosis is an important pathological hallmark of both ischemic and hemorrhagic stroke and can cause secondary functional decline adding to the deficit caused by the initial brain damage (Zille et al., 2017). Ferroptosis is therefore considered an important therapeutic target and may potentially serve as a novel biomarker as highlighted by Qu et al. through a combined *in silico* and *in vivo* approach. First, the authors studied transcriptome data from the Gene Expression Omnibus (GEO) database, identifying ferroptosis-related genes transcribed differently in ischemic stroke cases vs. controls. Additional Mendelian randomization analyses suggested a potential causal association between enoyl-coA hydrolase 1 (ECH1) and ischemic stroke (IS). ECH1 was downregulated in IS cases, which was confirmed in a rodent stroke model. ECH1 may therefore be an interesting biomarker or therapeutic target deserving closer investigation in future studies.

Ischemic stress may not only cause neuronal cell death but may also cause more subtle pathological alterations. Among those, damage to cytoskeletal elements has been considered as a major contributor to impaired neuronal function. The expression of microfibrillar-associated protein 5 (MFAP5) is reduced immediately after ischemic stress. Höfling et al. provide a spatial analysis of MFAP5 in the ischemic brain, employing different imaging techniques including fluorescence-based microscopy, confocal laser scanning microscopy, and 3D surface reconstruction. Although MFAP5 protein expression declined in the ischemic region, there was no clear spatial association with other cells such as micro- or astroglia, or the cerebral vasculature. However, the MFAP5 decline exhibited characteristics similar to those of neurofilament light chain (NfL) and microtubule-associated protein 2 decline, the latter being among the earliest proteins showing reduced expression under ischemic conditions. Thus, MFAP5 may not only have similarly decisive function in the neuronal cytoskeleton, but may also serve as a marker indicating neurodegeneration (declined expression) or neuroprotection (preserved expression), respectively.

Electroacupuncture is an increasingly popular treatment modality in China, combining both traditional and modern approaches. It is also used after IS, but any potential mechanisms of action(s) are only incompletely understood. In their work, Wu et al. focused on so-called “super-enhancers,” regulatory elements that control transcriptional programs being decisive for cellular responses to ischemic stress, and genes governed by their activity. The key finding was that electroacupuncture reduced the expression of HDAC7. HDAC7 is a class II histone deacetylase involved in cellular processes such as differentiation and cell survival. However, excessive HDAC7 expression may cause microvascular endothelial cell dysfunction, enhanced oxidative stress, inflammatory responses, and cell death. Wu et al. also identified other central transcriptional regulatory circuitries although their detailed impact will require further investigation.

Large animal models are increasingly recognized as a valuable step in translational research programs aiming at clinical implementation (Taha et al., 2022). The reasons for this comprise a more complex behavioral repertoire for large animal species, augmented experimental options, and brain anatomy closer to human patients, including a gyrencephalic cortex and higher white matter content (Boltze et al., 2017). Nevertheless, the application of recanalization therapies such as thrombolysis has so far been restricted to canine and non-human primate species for anatomical reasons, severely limiting the translational value of other, more widely available large animal models. Nowak et al. present a new porcine model overcoming this limitation, which is of particular value in the recanalization era. IS was induced by catheter-based injection of thrombin into the ascending pharyngeal artery supplying the rete mirabile. An imaging-guided approach enabled the prediction of the area affected by subsequent thrombosis, and IS was confirmed by cranial magnetic resonance imaging (MRI). Thrombolysis using recombinant tissue plasminogen activator (rtPA) was induced 2 h after IS induction and the resulting lesion was confirmed by MRI and histology 1 month later. The work also features *in vitro* data investigating rtPA thrombolytic activity in porcine blood clots. It is expected that this model will be of exceptional value for evaluating novel cerebroprotective therapies in the context of recanalization approaches, a major priority in current translational stroke research (Wechsler et al., 2023).

## Insights from clinical research

Vascular cognitive impairment and dementia are feared sequelae of IS, affecting up to one out of three stroke survivors. Targeted rehabilitation may help in mitigating the impact of such cognitive impairment, but early identification of patients at risk remains a major clinical challenge. While several biomarkers such as amyloid beta (Aβ), Tau, NfL, and glial fibrillary acidic protein (GFAP) as well as angiogenic factors (e.g., VEGF, Flt-1, Tie-2, PIGF, and FGF) have been identified as potentially associated with post-stroke dementia onset, they have not yet been clinically validated. Anil et al. present results from a clinical investigation comparing patients from Appalachian vs. non-Appalachian counties. People in Appalachian regions are an underserved population with higher chronic health conditions, IS incidence, and stroke-related mortality than other populations. Intracranial blood samples were obtained proximally and distally from the thrombus in patients undergoing thrombectomy, and analyzed using proteomic analysis. Arterial blood control samples were obtained from patients with non-acute cerebrovascular disease. Stroke patients exhibited higher levels of GFAP. In the Appalachian patient population, control patients also had significantly higher levels of Aβ40, Aβ42, and VEGF. In the non-Appalachian population, only GFAP was significantly different between stroke patients and controls. While this may point at a potential role of GFAP as an acute stroke biomarker, the differences between Appalachian and Non-Appalachian populations may require additional investigation, particularly considering socioeconomic and environmental factors.

Cerebral white matter hypointensities (WMH) are a hallmark of cerebral small vessel disease and an early indicator for cognitive impairment both in human patients and animal models (Staekenborg et al., 2008; Kaiser et al., 2014). Carotid intraplaque neovascularization (CINV) is a hallmark of plaque instability and positively associated with WMH burden (Pan et al., 2024). However, the longitudinal relationship between CINV and WMH progression is not well-described. Zhang et al. address this question in a longitudinal, imaging-based study. During a mean follow-up of 1.5 years, the authors found WMH progression in almost two thirds of patients. High-grade CINV was independently associated with deep WMH progression after correction for confounders such as frequent comorbidities and other vascular risk factors (odds ration >2). Thus, CINV may serve as both a biomarker and a therapeutic target for WMH progression.

Yuan et al. also applied advanced imaging methods to assess cerebral vascular wall fitness but for predicting intracranial aneurysm (IA) rupture. The retrospective bi-center study analyzed high-resolution vessel wall imaging datasets obtained from 422 IAs. These were used for machine learning (ML) model training, testing, and validation with both IA and parent artery (PA) walls being investigated. Numerous radiomic features were analyzed, and eight IA and four PA wall features were finally considered. Different ML models were tested against a standard radiological scoring system. IA rupture risk was reliably predicted by both the radiological scoring and ML models with the best model outperforming the radiological scoring in terms of diagnostic utility. As a non-invasive tool, selected ML methods may add benefit to IA diagnosis and rupture risk assessment after further clinical validation.

An often-neglected cause of neurovascular unit (NVU) impairment, myeloproliferative neoplasms (MPNs), was investigated by Kuznezova et al. in a small clinical pilot study enrolling 39 patients against 11 healthy controls. The team conducted a thorough analysis of baseline hemogram parameters, comorbidities and antiplatelet use before conducting functional MRI. The test paradigm was to squeeze a rubber ball at the frequency provided by a pulsing symbol on a screen presented to the subjects. Stronger activation of the right primary sensorimotor cortex in MPN patients was observed as compared to healthy controls. Higher platelet counts were typically seen in MPN patients and may represent a relevant confounder to control for in additional research and validation steps.

## Knowledge synthesis by narrative and systematic reviews

Arterial spin labeling (ASL) allows the investigation of cerebral blood flow (CBF) with the use of exogenous contrast agents in MRI and is increasingly used to assess CBF in health and brain disease (Huang et al., 2023). Habibi et al. review the state-of-the-art investigations of CBF and cerebrovascular autoregulation under hypoxia using ASL. The authors explain how ASL identifies signs of cerebrovascular (mal-)adaptation, impaired autoregulation, as well as early and progressing neurovascular unit dysfunction. They also discuss the diagnostic potential of ASL-derived metrics as biomarkers, especially for non-invasive longitudinal assessment of vascular fitness in patients and clinical populations at risk. Importantly, the authors contrast ASL against other imaging modalities pointing out the respective advantages and disadvantages. They conclude that ASL is a valuable tool to detect early pathological changes and may allow for precision diagnoses in conditions driven by acute and chronic hypoxia.

Modern cerebroprotective strategies have recently gained new momentum and are believed to hold therapeutic potential in both acute and chronic neurodegenerative diseases. Importantly, cerebroprotective approaches are no longer considered stand-alone treatments but should be seen in the context of established treatment approaches (Boltze and Fisher, 2026). Mongan and Sharma provide a narrative review putting the spotlight on neuritin, a gene encoding a glycosylphosphatidylinositol-anchored protein being highly conserved across vertebrate species. Neuritin plays an important role during neural development, contributes to synaptic plasticity and has been shown to exert robust cerebroprotective effects. The review particularly focuses on both activity-dependent and -independent molecular mechanisms governing neuritin activity, as well as specific cellular functions of neuritin. It also provides a very detailed overview of neuritin signaling pathways and downstream targets. Next to its physiological roles, the authors provide a detailed overview of neuritin actions under pathological conditions. This goes beyond cerebroprotection but also comprises immunoregulatory and angiogenic properties. Based on this cumulated knowledge, Mongan and Sharma provide detailed insights into therapeutic applications in acute and chronic neurodegenerative disorders. This comprehensive review may serve as a valuable compendium for all interested in the field of cerebroprotection.

Rithiely et al. systematically review and meta-analyze the therapeutic impact of non-invasive brain stimulation techniques, an emerging area in translational and clinical neuroscience. The specific focus was on the improvement of post-stroke motor impairment and disability. There were mixed results. Repetitive transcranial magnetic stimulation (TMS) was more often associated with improvements in general neurological function, with moderate to large effect sizes. Contrasting this, transcranial direct current stimulation (tDCS) exerted rather small effects on motor recovery. Remarkedly, certainty of evidence was very low to low across most studies and final result interpretation is impaired by methodological heterogeneity, reporting imprecision, and protocol variability. While the results are generally promising, more rigorous studies are required in the future to verify the beneficial effects reported so far.

## Summary and outlook

This Research Topic provides detailed and valuable insights into recent advances in the understanding of neurovascular and neurodegenerative disorder pathobiology as well as emerging diagnostic and therapeutic developments. It also points out potential knowledge gaps to be filled by future research in the field. Thus, it should be a valuable collection of contemporary research findings being of interest for the global neuroscience community.
